# Experiences and needs of family members of perinatal infant deaths: a meta-synthesis

**DOI:** 10.3389/fpubh.2025.1580039

**Published:** 2025-07-01

**Authors:** Nana Cui, Shasha Wu, Xiaoyun Wang, Lei Sheng

**Affiliations:** ^1^Neonatal Ward, Jining No.1 People's Hospital, Jining, Shandong, China; ^2^Nursing Department, Jining No.1 People's Hospital, Jining, Shandong, China

**Keywords:** experiences and needs, family members, perinatal infant, deaths, meta-synthesis

## Abstract

**Purpose:**

This meta-review of qualitative studies aims to explore the experiences and needs of family members following perinatal infant deaths.

**Design:**

A qualitative meta-synthesis was conducted.

**Data sources:**

Four databases, PubMed, CINAHL, EMBASE, and Web of Science, were searched from inception through November 2024. An initial search using the keywords “perinatal death”, “family members”, and “qualitative research” retrieved 496 articles. Based on predefined inclusion and exclusion criteria, 10 studies were selected for inclusion.

**Review methodology:**

The Critical Appraisal Skills Programme (CASP) Qualitative Research Checklist was used to assess the quality of included studies.

**Results:**

Ten studies from nine countries were analyzed, yielding five overarching themes: negative emotional reactions, searching for the cause of death, rebuilding of life, reconstruction of meaning, and need for support.

**Conclusion:**

Negative emotional responses are an inevitable and profound part of the bereavement process for families experiencing perinatal loss. Identifying the cause of death helps families understand their loss and reduces uncertainty and self-blame. Central to the grieving process is the reconstruction of emotional and psychological meaning, which involves redefining life's purpose and gradually restoring a sense of normalcy. However, many of these families' needs remain unmet. There is an urgent need for multidisciplinary strategies to provide comprehensive, tailored support.

## 1 Introduction

Perinatal mortality remains a critical global public health issue. According to the World Health Organization (1), perinatal death encompasses the period from 22 weeks of gestation through to the first 28 days post-birth. In 2019, an estimated two million babies [90% uncertainty interval (UI) 1.9–2.2] were stillborn at 28 weeks of gestation or later, with a global stillbirth rate of 13.9 per 1,000 total births (90% UI 13.5–15.4) (2). Studies from Europe, Australia, and the United States have shown that perinatal loss not only imposes significant emotional and psychological burdens on families but also contributes to increased societal and healthcare costs (3–5). For mothers and their families, the confirmation of fetal death is a psychologically traumatic event with long-term effects extending beyond emotional distress to physical and social dimensions (6). Fathers too, experience substantial emotional trauma, often intensified by witnessing their partner's suffering (7, 8).

Despite international consensus guidelines clearly advocating comprehensive family support for perinatally bereaved families (9), gaps persist between the emotional support needs of bereaved parents and the care provided in clinical practice. Research highlights that the quality of bereavement care significantly influences how parents cope with and recover from their loss (10, 11). However, parents frequently report dissatisfaction with the offered support, citing inadequate healthcare resources and insufficient attention to individual emotional needs (11). Current bereavement care often focuses primarily on physical aspects, neglecting the systematic integration of tailored psychosocial support (10, 12). This incongruity underscores the urgent need to re-evaluate support frameworks by critically analyzing lived experiences. A nuanced understanding of lived experiences is essential in informing effective, empathetic perinatal bereavement interventions. Meta-synthesis offers a valuable method for synthesizing qualitative evidence, allowing for a deeper exploration of complex emotional and psychological processes (10, 13). Qualitative research is particularly well-suited to capturing the multidimensional emotional trajectories and the dynamic process of the bereaved and providing a contextualized framework for clinical practice.

Therefore, this meta-synthesis aims to analyze, interpret, and integrate existing qualitative research on the experiences and needs of families affected by perinatal infant deaths. It seeks to answer the following questions: (1) What are the experiences and needs of family members whose infants die during the perinatal period? (2) What recommendations can be drawn from these studies to inform clinical practice, education, and future research to better support families experiencing perinatal loss?

## 2 Methods

### 2.1 Research design

Meta-synthesis is a well-established methodology for integrating qualitative findings in health services research (14–16). It serves as an umbrella term for the synthesis of results derived from various qualitative methods and is commonly used to interpret and contextualize qualitative evidence (17). This methodology allows for a deep understanding of participants' meanings, experiences, and perspectives, made possible by the qualitative nature of the included studies, and a broad synthesis by incorporating findings across diverse healthcare settings and populations (18).

### 2.2 Search strategy

To identify relevant literature, four electronic databases, PubMed, CINAHL, EMBASE, and Web of Science, were searched from their inception to 30 November 2024. Articles were screened based on predefined inclusion and exclusion criteria formulated using the Population, Intervention, Comparison, Outcomes, and Study (PICOS) framework (19). The search was limited to articles in English or Chinese. A detailed search strategy is provided in Supplementary Table 3. This meta-synthesis followed the Preferred Reporting Items for Systematic Reviews and Meta-Analyses (PRISMA) guidelines. Additionally, the reference lists of the included articles were manually reviewed to identify further eligible studies.

### 2.3 Study selection

**Figure 1:**
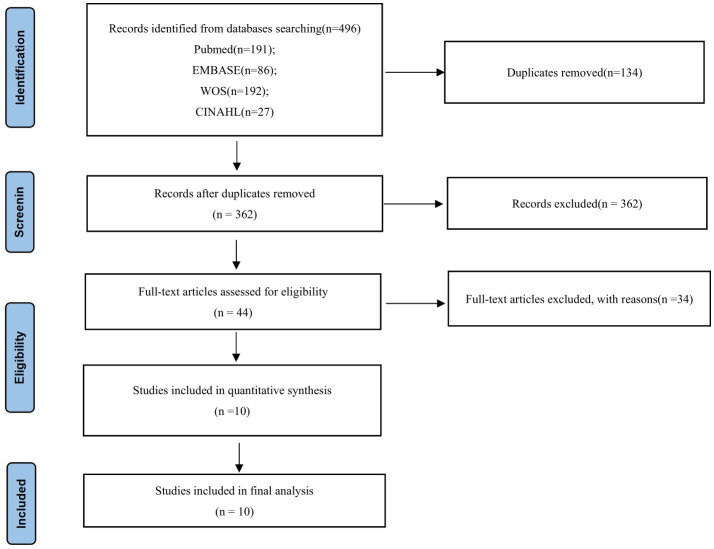
Flow chart.

All search results were imported into EndNote, which was also used to import articles and remove duplicates. Two researchers independently screened titles and abstracts using the inclusion and exclusion criteria, followed by a full-text review to confirm eligibility (Figure 1).

The inclusion criteria were as follows: (a) The study population included parents who had experienced a perinatal death (fetal/neonatal deaths between the 22nd week of gestation and the 28th day after birth); (b) The study focused on parental experiences needs to be related to perinatal deaths; (c) The context was explicitly related to perinatal deaths; (d) The research design was qualitative, using methods such as phenomenology, ethnography, grounded theory, hermeneutics, narrative analysis, or thematic analysis and primarily analyzed textual data rather than digital data; (e) The publication timeframe extended from database inception to 30 November 2024; and (f) The articles were peer-reviewed and published in English or Chinese. Exclusion criteria were as follows: (a) Conference abstracts, dissertations, or gray literature; (b) Studies from which qualitative data could not be extracted; and (c) Articles with full text literature was unavailable through PubMed LinkOut, LibGen, and the host institution's interlibrary loan system.

### 2.4 Quality assessment

All included studies were assessed using the Critical Appraisal Skills Programme (CASP) Qualitative Checklist, which assesses whether each included study had: A clearly stated aim; An appropriate methodology and study design; A suitable recruitment strategy; Sound data collection and ethical considerations, Rigorous data analysis; Clear presentation of findings; and overall research value. Two researchers (CNN and WXY) independently reviewed each study based on a checklist. In case of disagreement, a third researcher (WSS) facilitated consensus through discussion. Two such disagreements arose during the review and were resolved through this collaborative process. A quality assessment form was created (Table 1). To enable comparison across studies. This table summarized key quality indicators, including the purpose of the study, appropriate qualitative methods, study design, recruitment strategies, data collection methods, the relationship between the researcher and the participants, consideration of ethical issues, data analysis rigor, findings presentation, and the value of the study.

**Table 1:** Use of the critical assessment screening programme (CASP).

**No**.	**Author/Year**	**CASP-items**									
		**1**	**2**	**3**	**4**	**5**	**6**	**7**	**8**	**9**	**10**
1	Downe et al. (2013)	Y	Y	Y	Y	Y	C	Y	Y	Y	Y
2	Camacho-Ávila et al. (2019)	Y	Y	Y	Y	Y	C	Y	Y	Y	Y
3	Lizcano et al. (2019)	Y	Y	Y	Y	Y	C	Y	Y	Y	Y
4	Kuforiji et al. (2024)	Y	Y	Y	Y	Y	C	Y	Y	Y	Y
5	Zheng et al. (2024)	Y	Y	Y	Y	Y	C	Y	Y	Y	Y
6	Azeez et al. (2022)	Y	Y	Y	Y	Y	C	Y	Y	Y	Y
7	Kavanaugh et al. (2005)	Y	Y	Y	Y	Y	C	Y	Y	Y	Y
8	Sutan et al. (2012)	Y	Y	Y	Y	Y	C	Y	Y	Y	Y
9	Arach et al. (2022)	Y	Y	Y	Y	Y	C	Y	Y	Y	Y
10	Horey et al. (2012)	Y	Y	Y	Y	Y	C	Y	Y	Y	Y

1. Was there a clear statement of the aims of the research? 2. Is a qualitative methodology appropriate? 3. Was the research design appropriate to address the aims of the research? 4. Was the recruitment strategy appropriate to the aims of the research? 5. Was the data collected in a way that addressed the research issue? 6. Has the relationship between researcher and participants been adequately considered? 7. Have ethical issues been taken into consideration? 8. Was the data analysis sufficiently rigorous? 9. Is there a clear statement of findings? 10. How valuable is the research? Y, yes; C, can't tell; N, no.

### 2.5 Data extraction and analysis

Data were extracted independently by two researchers (WXY, SL), including and included authors, year of publication, sample size, age and sex of participants, time of death, newborn age, country of publication, study design and analytical method. The extracted information was compiled in tabular form and cross-checked by a third researcher (WSS). Thematic synthesis followed the framework proposed by Thomas and Harden (24). First, the primary studies were systematically reviewed and iteratively read by CNN and WXY to generate Initial codes. Second, SL and WSS grouped these codes into descriptive themes by comparing their similarities. Finally, CNN refined these themes to identify overarching analytical constructs, generating new insights and hypotheses. These analytical themes were supported by key findings from the original articles, providing a more comprehensive synthesis of the research field. Any discrepancies encountered during the process were resolved through discussions in group meetings.

## 3 Results

### 3.1 Record search results

The study selection process is illustrated in Figure 1. The initial search identified 496 articles, of which 134 duplicates were removed using EndNote software. After title and abstract screening, 362 articles were reviewed, and 318 were excluded based on predefined inclusion and exclusion criteria. Subsequently, 44 full-text articles were assessed, and 34 were excluded for the following reasons: (1) non-qualitative study design (*n* = 15); (2) focus on healthcare providers' perspectives rather than family members (*n* = 8); (3) lack of extractable qualitative data (*n* = 6); and (4) duplicate study populations or contexts already represented in included studies (*n* = 5). Finally, 10 full-text articles met the eligibility criteria and were included in the quality assessment and synthesis.

### 3.2 Quality assessment of included studies

All 10 included studies fulfilled at least nine out of the 10 criteria on the Critical Appraisal Skills Programme (CASP) qualitative checklist, indicating robust methodological quality. Consequently, all 10 studies were retained for the final qualitative synthesis.

### 3.3 Basic characteristics of the included studies

The 10 studies employed three principal qualitative methodological approaches: phenomenological design (*n* = 5), exploratory-descriptive design (*n* = 3), generic qualitative methodology (*n* = 2). Analytical methods included inductive thematic analysis and content thematic analysis (Table 2).

**Table 2:** Basic characteristics of qualitative studies included in the meta-synthesis.

**Author/Year**	**Country**	**Research design**	**Data analysis**
Downe et al. (2013)	Britain	Phenomenological study design	Inductive thematic analysis
Camacho-Ávila et al. (2019)	Spain	Hermeneutic phenomenology design	Inductive thematic analysis
Lizcano et al. (2019)	Colombia	Descriptive phenomenological design	Inductive thematic analysis
Kuforiji et al. (2024)	Nigeria	Hermeneutic phenomenology design	Inductive thematic analysis
Zheng et al. (2024)	China	Interpretivist constructionist phenomenology	Inductive thematic analysis
Azeez et al. (2022)	Australia	Descriptive, exploratory research	Inductive thematic analysis
Kavanaugh et al. (2005)	America	Descriptive phenomenologic approach	Inductive thematic analysis
Sutan et al. (2012)	Malaysia	Qualitative, exploratory and descriptive analysis	Inductive thematic analysis
Arach et al. (2022)	Uganda	Qualitative study	Content thematic analysis
Horey et al. (2012)	Australia	Qualitative study	Content thematic analysis

### 3.4 Demographic characteristics of study participants

Sample sizes in the included studies ranged from 10 to 32 participants (Table 3). The study populations consisted of family members of neonates who had died, aged between 18 and 63 years, with 125 female and 59 male participants. All 10 studies reported the time since death, ranging from 5 weeks to 12 years. The studies were conducted in a variety of countries: Australia (*n* = 2), the United Kingdom (*n* = 1), Spain (*n* = 1), Colombia (*n* = 1), Nigeria (*n* = 1), China (*n* = 1), the United States (*n* = 1), Malaysia (*n* = 1), and Uganda (*n* = 1). All 10 studies were published between 2005 and 2024.

**Table 3:** Demographic characteristics of individual study participants included in the meta-synthesis.

**Author/Year**	**Sample size**	**Age of participants**	**Sex**	**Time since loss**	**Age of baby**
Downe et al. (2013)	*n* = 25	18–44	Female = 19 Male = 6		24–42 weeks
Camacho-Ávila et al. (2019)	*n* = 21	26–43	Female = 13 Male = 8	3 months−5 years	24 weeks−6 days
Lizcano et al. (2019)	*n* = 15	18–54	Female = 0 Male = 15		22–38 weeks
Kuforiji et al. (2024)	*n* = 14	22–41	Female = 14 Male = 0	< 5 years	< 28 days
Zheng et al. (2024)	*n* = 28	32.96 ± 4.97	Female = 28 Male = 0	< 1 years	≥24 weeks
Azeez et al. (2022)	*n* = 10	31–42	Female = 0 Male = 10	1–12 years	30 min−27 days
Kavanaugh et al. (2005)	*n* = 23	>18	Female = 17 Male = 6	5–21 weeks	26, 27 weeks
Sutan et al. (2012)	*n* = 16	23–37	Female = 16 Male = 0	6–12 months	26–39 weeks
Arach et al. (2022)	*n* = 32	17–68	Female = 18 Male = 14	< 2 years	≥28 weeks
Horey et al. (2012)	*n* = 17		Female = 14 Male = 3	< 7 years	

### 3.5 Key findings of the meta-synthesis

Thematic analysis of 33 key quotes from the 10 included studies generated five overarching themes: negative emotional reactions, searching for the cause of death, rebuilding life, reconstruction of meaning, and need for support (Table 4).

**Table 4:** Supporting quotes as related to five themes.

**Author/Year**	**Supporting quotes underpinning the five themes**				
	**Emotional outpouring**	**Finding cause of death**	**Need for support**	**Meaning reconstruction**	**Rebuilding life**
Downe et al. (2013)	Beyond distress: Bowled over by the horror…	Expressed a strong drive to find out why their baby died.	Filling the gap someone to help you and guide you…	Making irretrievable moments precious	Positive caring in the care: ‘they hold a special place in our lives
Camacho-Ávila et al. (2019)	The shock of losing a baby and the pain of giving birth to a stillborn baby	Not knowing the cause of death or not having a clear explanation about the causes.	Receiving individualized care from midwives and physicians could become the most important source of comfort for parents.	“We have had a baby.”	Saying goodbye to the deceased baby, having the baby's footprint, keeping the memory of the baby alive
Lizcano et al. (2019)	It leaves him devastated, in pain, and feeling shattered.		To give support and strength to their wives or partners	Finding Meaning in Loss	By acknowledging and giving a special meaning to the presence of the deceased child in their lives and home.
Kuforiji et al. (2024)	Caused feeling of anxiety and uncertainty.		Mothers expected comprehensive emotional support from health care professionals		In reassuring mothers of hope for future pregnancies and living babies.
Zheng et al. (2024)	Restrained expressions of grief.		Receiving unexpected levels of help, understanding, an support from family, friends, and colleagues	Reshaping beliefs and views about life and death.	Gained a renewed sense of purpose and direction and felt motivated to make positive changes in their lives.
Azeez et al. (2022)	A complicated grief experience	Disbelief at neonatal death outcome	Disenfranchised grief: lack of social recognition and acknowledgement	It challenging to balance expressing their grief with a desire to support others and attend to responsibilities	
Kavanaugh et al. (2005)	Feeling intense emotions after the death	Parents tried to make sense of their loss and determine why it occurred	Feeling abandoned or unsupported.Seeking diversions and support	Creating and Cherishing Memories of their Infant	Contemplating future pregnancies
Sutan et al. (2012)	Confusion Feeling of emptiness Anger Anger Anxiety over subsequent pregnancy	Parents tried to make sense of their loss and determine why it occurred.	Support during grief.	By practicing religious activities they were able to reduce the pain they suffered and make their mind more accepting of the situation.	
Arach et al. (2022)	Pain, confusion, facing multiple challenging roles, concern about the health of the partners, health care providers' reaction, blame and guilt.	Women were commonly blamed by their partners and in-laws for the perinatal deaths. This could be because family members were searching for the cause of the perinatal deaths.	Family and community support		
Horeyet al. (2012)	Hazy, emotionally wrecked My biggest fear, and that's what a lot of people would have about it, is ‘oh shit, I might have done something wrong and now I'm going to be blamed.	Because I'm not asking questions and I don't have the what if. And even though it was unexplained I did everything I could to find out for myself.	Your doctor makes other decisions for you like. They are quite happy to make those decisions and guide you so why is suddenly when things go wrong... everyone backs off.	It was like another way of reconnecting with him. You get such a level of detail about your child that you wouldn't get in a different setting.	The autopsy did explain the death and gave the parents new information to consider when planning for another baby.

### 3.6 Negative emotional reactions

Perinatal loss evokes complex and intense emotional responses. Mothers who experienced stillbirths often reported feelings of regret and self-blame, attributing the loss to delayed care-seeking, lack of resources, or choosing to give birth at home (20). The diagnosis of stillbirth during antenatal care was associated with prolonged anxiety and emotional distress (21). Most participants blamed themselves for the outcome, resulting in deep guilt and psychological trauma (22). The death of a newborn was described as one of the most devastating experiences in a parent's life, with distress persisting for years (23). Unlike other types of bereavement, perinatal loss was associated with not only emotional pain but also physical symptoms such as sleep disturbances, nightmares, tingling, and somatic pain (24). Family members reported feelings of shock, uncertainty, loneliness, loss, guilt, self-blame, and anger (25–27). The confirmation of a newborn's death marked the beginning of an emotionally taxing grieving process, often worsened by a lack of communication and support from healthcare providers (27). Almost all mothers mentioned intense loneliness following the loss (28), especially when the death was sudden and unexpected, leading to profound feelings of helplessness and disbelief (23, 25, 26). Some women internalized blame for the loss, and the absence of empathetic communication from medical professionals deepened their isolation (28, 29). This isolation extended beyond emotional loneliness, encompassing confusion about the future and shaken sense of identity.

### 3.7 Finding the cause

Parents expressed a strong desire to understand the cause of their infant's death (26, 29). In seeking explanations, some fathers compared their experiences with those of family and friends, revisiting medical decisions and evaluating the healthcare system's response (23). The need for support and support in understanding the cause was especially acute (20). In many cases, women were blamed by their partners and in-laws, reflecting a broader cultural need to assign responsibility (20). Parents also sought meaning through philosophical or spiritual interpretations of death (26). Autopsies were highlighted as the most definitive method for establishing the cause of death, especially in cases of stillbirths (30–32). However, parents needed detailed, compassionate explanations about autopsy procedures, free from blame, and delivered within a supportive and trusting healthcare environment (32). Across both stillbirth and neonatal death cases, transparent communication from healthcare providers was a recurring need.

### 3.8 Need for support

Participants frequently reported that not all healthcare professionals were adequately trained to communicate compassionately about perinatal death (28). Mothers desired comprehensive emotional support from healthcare professionals, including opportunities to discuss their feelings openly with doctors and midwives (21). Personalized care from these professionals was seen as particularly comforting (27). Parents reported that they would have coped better if professionals had communicated more transparently during hospitalization (29). In addition to professional support, emotional support from family and friends played a critical role in coping. Parents appreciated those who remained present and comforting after the loss and expressed a desire for continued support even after the funeral (20, 26). Many fathers reported difficulty balancing their own grief with their perceived responsibilities to support others (23). Many participants also described engaging in activities to stay busy, distract themselves, and seek social support.

### 3.9 Reconstruction of meaning

Many participants reported that perinatal loss caused them to question the meaning of life. Some sought to reconstruct meaning through reflective practices, spiritual interpretations, and remembrance (33). Creating and conserving memories of the deceased child, such as through keepsakes or rituals, was seen as an important way to cope (29). The experience of loss brought about a shift in participants' worldviews. Parents learned to integrate the child's memory into their ongoing lives and identities, sometimes perceiving the deceased child as a continued spiritual presence (25). Others processed the trauma by making sense of the event and reevaluating their lives in its aftermath (22).

### 3.10 Rebuilding lives

Perinatal bereavement often leads to a re-evaluation of life priorities and values. Some fathers expressed gratitude for the experience of fatherhood, however brief, and described how the loss had deepened their appreciation for loved ones. When fathers give meaning to perinatal death and acknowledge the spiritual presence of their children in their lives, they can move beyond the pain of losing their children (25). Through meaning-making and spiritual reflection, some parents were able to move forward, regaining a sense of purpose and direction (22). The birth of a healthy child after the loss was often seen as part of the healing process, helping parents recover from grief and renew hope (26).

## 4 Discussion

This review synthesizes findings from 10 qualitative studies on the experiences and needs of families affected by neonatal death. It provides valuable insights that confirm and extend existing knowledge on this important topic.

### 4.1 Negative emotional reactions

Perinatal bereavement is a deeply traumatic experience, often marked by grief, guilt, and somatic symptoms. These reactions align with established theories of traumatic loss. Stroebe and Schut's Dual Process Model (1999) (34) provides a theoretical framework to understand the oscillation between loss-oriented and recovery-oriented coping strategies observed in the included studies. Parents often experience profound distress compounded by feelings of loneliness, lack of information, guilt, and self-blame. Unlike other forms of bereavement, perinatal loss represents not just the physical absence of a child, but the chattering of future hopes and imagined lives. Many mothers internalize blame, attributing the death to their actions or decisions, which can lead to lasting psychological trauma. Cultural differences also shape grief expression. Whereas, Western studies emphasize individualized grief (35), participants in Nigeria and Uganda described a communal grief burdened by stigma (20). These findings highlight the importance of culturally sensitive bereavement care. In low-resource settings, structural inequalities often intensify parental guilt, reinforcing the need for context-specific bereavement protocols as advocated by the International Stillbirth Alliance Conference (36, 37). Timely, compassionate communication and the provision of clear information are critical. Healthcare professionals should engage with parents early following a diagnosis of stillbirth, offering both informational and emotional support.

### 4.2 Finding the cause

Understanding the cause of death is a central need for bereaved parents. This review suggests that perinatal bereavement not only causes profound emotional trauma but also triggers a deep need to understand why the death occurred. For many parents, discovering the cause becomes one of their most urgent concerns following the loss (26, 29). The “search for meaning” aligns with Neimeyer's constructivist theory of grief, which emphasizes the importance of sense-making in adapting to loss (38). Parents' intense need to understand the cause of their infant's death often includes intense questioning, self-reflection, particularly by fathers, internal attribution of responsibility within the family, and seeking explanations through philosophical and religious perspectives. These reactions reflect not only emotional needs but also reveal the importance of social and cultural factors in dealing with such events. For parents, this need is not only motivated by rational curiosity, but also to find a way to process and accept reality. Understanding the exact cause helps parents reduce feelings of self-blame and uncertainty, focus their grief, and inform future reproductive decisions.

### 4.3 The need for support

Perinatal bereavement is a complex and multi-layered experience that impacts not only individual mental health but also the wider family and social support network. Research has shown that the quality of care and support received before and after an infant's premature death is a critical factor in parents' immediate and long-term wellbeing (39). This support must extend beyond medical care to include emotional, social, and psychological dimensions. Nurses and midwives play a vital role in post-bereavement care and are frequently cited as a primary source of support (40). Their presence, active listening, and emotional responsiveness are key to helping parents navigate their grief. Beyond hospital settings, community-based services, such as primary care clinics and outreach programmes, are critical in providing ongoing support (30, 39). These community resources can provide both long-term emotional support and practical help as families gradually recover.

Peer support consistently emerges as valued source of comfort and strength. In line with the Sands International Bereavement Care Guidelines (2023) (41), peer networks are recommended as adjuncts to clinical care. Parents often report that connecting with others who have had similar experiences provides affirmation, comfort, hope, and a sense of shared understanding (42, 43). Such support reduces feelings of isolation and helps parents feel less alone in their grief. Personalized care also plays a crucial role in the healing process. Community organizations and professional bodies can provide training and educational resources to help family members better support bereaved families. Healthcare professionals must be equipped with the skills to offer empathetic and culturally sensitive care. However, some relatives may lack sufficient knowledge or skills to engage, which can increase parental isolation and distress (44). Raising public awareness and improving communication skills across the community is therefore essential to ensure that support systems do not inadvertently contribute to feelings of abandonment.

### 4.4 Reconstruction of meaning

While perinatal bereavement brings profound sorrow, it can also offer an opportunity for families to reframe and redefine their lives. This review shows that, despite overwhelming grief, many parents strive to reconstruct meaning and redefine their values and goals. Acts such as preserving fond memories, collecting meaningful mementoes, reframing life perspectives, and engaging in deep reflection help parents rediscover purpose amid their loss. Parents often continue to acknowledge the presence of their deceased child in their life and family and imbue that presence with lasting meaning. This aligns with Tedeschi and Calhoun's (45) concept of post-traumatic growth, a process of meaning-making through memorialization and spiritual reframing parallels. Additionally, participants' efforts to integrate the deceased child into family narratives resonate with Klass's (46) theory of continuing bonds, challenging earlier grief models that prioritized detachment. Rather than a simple reflection on the past, this process involves reimagining life and forging a future shaped by the child's enduring influence. Such meaning-making may improve psychological resilience and strengthen family relationships. Many mothers, for example, choose to journal about their thoughts and feelings, allowing them to explore how the experience has transformed them (47). As young women find meaning in their experiences and gain new insights into their loss, the support provided during follow-up may undergo a critical shift. Tools such as the Perinatal Bereavement Care Checklist (48), which incorporates cultural and spiritual assessments into care planning, can help clinicians offer more sensitive and personalized support.

### 4.5 Rebuilding lives

Perinatal loss is often one of the most devastating experiences a family can endure, consistent with prior research (25). Yet, this review suggests that such loss can also become a catalyst for personal growth and renewed purpose, in line with Bonanno's resilience theory (49). The emotional upheaval caused by perinatal death can lead to a reassessment of life priorities, fostering greater gratitude, self-awareness, and motivation for positive change. This emotional shift exemplifies the ability of humans to find and appreciate the good in life despite the grief they face. When family members are able to assign meaning to the loss and acknowledge the spiritual presence of the child in their lives, they are often better equipped to cope with grief and embrace life anew. Practices such as meditation, prayer, or other forms of spiritual practice offer valuable coping resources, helping families face life's uncertainties with greater openness and resilience. For many families, a subsequent healthy pregnancy represents a key step in the healing process. It can restore hope and vitality while easing the lingering pain of loss. However, healthcare professionals must be sensitive to acknowledge psychosocial impact of prior loss and support parents accordingly (50). This includes grief education and training to ensure that professionals can respond with both competence and compassion during and after bereavement.

### 4.6 Strengths and limitations

The strengths of this study lie in its systematic approach to data retrieval and synthesis, as well as the international representativeness of the included studies, which enhance the reliability and relevance of the findings. A comprehensive literature search and rigorous data analysis helped ensure the review's credibility. However, some limitations must be acknowledged. The inclusion of only English and Chinese publications may have introduced language bias, and restricting the review to published studies could have excluded relevant gray literature. Additionally, nine of the ten included studies were conducted in Western countries, which limits the generalisability of the findings to other countries with different cultural backgrounds and healthcare systems. The relatively small number of studies included (*n* = 10) may also reflect publication bias or gaps in qualitative research on perinatal bereavement in non-Western contexts. Future research should explore the experiences and needs of mothers and mothers separately, as the grief responses, coping strategies, and support needs can differ significantly between parents. This would help inform more targeted and effective interventions.

## 5 Conclusion

Negative emotional reactions are an inevitable part of perinatal bereavement. Understanding the cause of the infant's death is fundamental need for parents, as it provides clarity, reduces self-blame, and supports emotional processing. Equally vital is the reconstruction of meaning on an emotional and psychological level, which involves redefining the meaning of life, finding new purpose and direction, and gradually restoring normality to life. Despite the profound impact of such losses, many needs of bereaved families remain unmet. There is an urgent need for multidisciplinary strategies to provide comprehensive support during and after bereavement.

### 5.1 Implications

Firstly, policymakers should develop a standardized and systematic bereavement care protocols to ensure coordinated support for affected families. This includes transparent communication regarding autopsy procedures, timelines, and results. Genetic counseling should be made available to assess future pregnancy risks. Psychological support services, including individual counseling, group therapy and support groups, should be accessible, and health education resources should be developed to enhance parents' knowledge and health literacy related to perinatal bereavement through health talks, pamphlets, and online resources.

Secondly, healthcare teams should include doctors, nurses, psychologists, and social workers, working collaboratively to provide all-round support to families. Specialized training should be provided to nurses and other healthcare professionals to help them acquire skills in communicating with bereaved families, and members of the team should communicate with each other on a regular basis to ensure information sharing and service coordination. Training should include psychological foundations, grief counseling and cultural sensitivity. Post-discharge outreach and partnerships with local community organizations can extend support beyond clinical settings.

Thirdly, healthcare professionals should adopt a personalized care approach that respects unique emotional needs and preferences. Compassionate communication, attentive listening, and flexible support are essential. Respecting parents' emotional expressions and personal choices ensures that they feel valued and understood. Healthcare professionals should demonstrate empathy and support in their interactions with families, listening to them, responding to their specific needs, and helping them find strength and direction in their grief. Nurses, social workers, occupational therapists, and psychologists should provide targeted interventions, which can be done through personalized assessment, whereby each family is assessed in detail to understand their psychological state, social support network and specific needs. Personalized assessments can guide the development of care plans, while continued contact, through regular home visits, telephone counseling, or online support, can help cope and recover over time.

### 5.2 Recommendations for future research

Perinatal bereavement is both a deeply personal tragedy and a challenge to healthcare systems and communities. Strengthening professional training, improving communication of medical information, establishing a multidisciplinary support system, and fostering the joint participation of all sectors of society are key to supporting grieving families. Future research should continue to explore the complexities of perinatal bereavement and contribute to the evidence base for effective, culturally responsive interventions. In doing so, we can ensure that no family faces this experience without hope or support.

## Data Availability

The datasets presented in this study can be found in online repositories. The names of the repository/repositories and accession number(s) can be found in the article/Supplementary material.
