# Supplementary material for: Experiences and needs of family members of perinatal infant deaths: a meta-synthesis
Source: Front Public Health. 2025 Jul 1;13:1580039. doi: 10.3389/fpubh.2025.1580039 (PMC12261921; doi:10.3389/fpubh.2025.1580039)
Supplement: Supplementary file 1 [file Table_1.docx]

**Identification**

Records identified from databases searching(n=496)

Pubmed(n=191);

EMBASE(n=86);

WOS(n=192);

CINAHL(n=27)

Duplicates removed(n=134)

Records excluded(n = 362)

**Screening**

Records after duplicates removed

(n = 362)

Full-text articles excluded, with reasons(n =34)

Full-text articles assessed for eligibility

(n = 44)

**Eligibility**

Studies included in quantitative synthesis

(n =10)

**Included**

Studies included in final analysis

(n = 10)

Fig 1. Flow chart
