# Supplementary material for: Experiences and needs of family members of perinatal infant deaths: a meta-synthesis
Source: Front Public Health. 2025 Jul 1;13:1580039. doi: 10.3389/fpubh.2025.1580039 (PMC12261921; doi:10.3389/fpubh.2025.1580039)
Supplement: Supplementary file 2 [file Table_2.docx]

| Table 1 Use of the Critical Assessment Screening Programme(CASP) | | | | | | | | | | | |
| --- | --- | --- | --- | --- | --- | --- | --- | --- | --- | --- | --- |
| No. | Author/Year | CASP-Items | | | | | | | | | |
|  |  | 1 | 2 | 3 | 4 | 5 | 6 | 7 | 8 | 9 | 10 |
| 1 | Downe et al.(2013) | Y | Y | Y | Y | Y | C | Y | Y | Y | Y |
| 2 | Camacho-Ávila et al.(2019) | Y | Y | Y | Y | Y | C | Y | Y | Y | Y |
| 3 | Lizcano et al.(2019) | Y | Y | Y | Y | Y | C | Y | Y | Y | Y |
| 4 | Kuforiji et al.(2024) | Y | Y | Y | Y | Y | C | Y | Y | Y | Y |
| 5 | Zheng et al.(2024) | Y | Y | Y | Y | Y | C | Y | Y | Y | Y |
| 6 | Azeez et al.(2022) | Y | Y | Y | Y | Y | C | Y | Y | Y | Y |
| 7 | Kavanaugh et al.(2005) | Y | Y | Y | Y | Y | C | Y | Y | Y | Y |
| 8 | Sutan et al.(2012) | Y | Y | Y | Y | Y | C | Y | Y | Y | Y |
| 9 | Arach et al.(2022) | Y | Y | Y | Y | Y | C | Y | Y | Y | Y |
| 10 | Horeyet al.(2012) | Y | Y | Y | Y | Y | C | Y | Y | Y | Y |

1. Was there a clear statement of the aims of the research?

2. Is a qualitative methodology appropriate?

3. Was the research design appropriate to address the aims of the research?

4. Was the recruitment strategy appropriate to the aims of the research?

5. Was the data collected in a way that addressed the research issue?

6. Has the relationship between researcher and participants been adequately considered?

7. Have ethical issues been taken into consideration?

8. Was the data analysis sufficiently rigorous?

9. Is there a clear statement of findings?

10. How valuable is the research?

Legend: Y = yes; C = can't tell; N = no.

| Table 2 Basic characteristics of qualitative studies included in the meta-synthesis | | | |
| --- | --- | --- | --- |
| Author/Year | Country | Research design | Data analysis |
| Downe et al.（2013） | Britain | Phenomenological Study design | Inductive Thematic Analysis |
| Camacho-Ávila et al.（2019） | Spain | Hermeneutic phenomenology design | Inductive Thematic Analysis |
| Lizcano et al.（2019） | Colombia | Descriptive phenomenological design | Inductive Thematic Analysis |
| Kuforiji et al.（2024） | Nigeria | Hermeneutic Phenomenology design | Inductive Thematic Analysis |
| Zheng et al.（2024） | China | Interpretivist Constructionist Phenomenology | Inductive Thematic Analysis |
| Azeez et al.（2022） | Australia | Descriptive, exploratory research | Inductive Thematic Analysis |
| Kavanaugh et al.（2005） | America | Descriptive phenomenologic approach | Inductive Thematic Analysis |
| Sutan et al.（2012） | Malaysia | Qualitative, exploratory and descriptive analysis | Inductive Thematic Analysis |
| Arach et al.（2022） | Uganda | Qualitative study | Content Thematic Analysis |
| Horeyet al.(2012) | Australia | Qualitative study | Content Thematic Analysis |

| Table 3 Demographic characteristics of individual study participants included in the meta-synthesis | | | | | |
| --- | --- | --- | --- | --- | --- |
| Author/Year | Sample size | Age of participants | Sex | Time since loss | Age of baby |
| Downe et al.（2013） | n=25 | 18-44 | Female= 19 Male= 6 |  | 24-42weeks |
| Camacho-Ávila et al.（2019） | n=21 | 26-43 | Female=13  Male= 8 | 3months-5years | 24weeks-6days |
| Lizcano et al.（2019） | n=15 | 18-54 | Female=0  Male=15 |  | 22-38weeks |
| Kuforiji et al.（2024） | n=14 | 22-41 | Female= 14 Male= 0 | <5years | <28days |
| Zheng et al.（2024） | n=28 | 32.96± 4.97 | Female= 28 Male= 0 | <1years | ≥24weeks |
| Azeez et al.（2022） | n=10 | 31-42 | Female=0  Male= 10 | 1-12years | 30 min-27days |
| Kavanaugh et al.（2005） | n=23 | >18 | Female= 17 Male= 6 | 5-21weeks | 26.27weeks |
| Sutan et al.（2012） | n=16 | 23-37 | Female= 16 Male= 0 | 6-12months | 26-39weeks |
| Arach et al.（2022） | n=32 | 17-68 | Female= 18 Male= 14 | <2years | ≥28w |
| Horeyet al.(2012) | n=17 |  | Female= 14 Male= 3 | <7years |  |

| Table 4 Supporting quotes as related to five themes | | | | | |
| --- | --- | --- | --- | --- | --- |
| Author/Year | Supporting quotes underpinning the five themes | | | | |
|  | Emotional outpouring | Finding cause of death | Need for support | Meaning reconstruction | Rebuilding life |
| Downe et al.（2013） | Beyond distress:Bowled over by the horror… | expressed a strong drive to find out why their baby died. | Filling the gap someone to help you and guide you… | Making irretrievable moments precious | Positive caring in the care:‘they hold a special place in our lives |
| Camacho-Ávila et al.（2019） | the shock of losing a baby and the pain of giving birth to a stillborn baby | not knowing the cause of death or not having a clear explanation about the causes. | receiving individualized care from midwives and physicians could become the most important source of comfort for parents. | “We have had a baby.” | Saying goodbye to the deceased baby,having the baby’s footprint, keeping the memory of the baby alive |
| Lizcano et al.（2019） | it leaves him devastated, in pain, and feeling shattered. |  | to give support and strength to their wives or partners | Finding Meaning in Loss | by acknowledging and giving a special meaning to the presence of the deceased child in their lives and home. |
| Kuforiji et al.（2024） | caused feeling of anxiety and uncertainty. |  | Mothers expected comprehensive emotional support from health care professionals |  | in reassuring mothers of hope for future pregnancies and living babies. |
| Zheng et al.（2024） | Restrained expressions of grief. |  | receiving unexpected levels of help, understanding, an support from family, friends, and colleagues | Reshaping beliefs and views about life and death. | gained a renewed sense of purpose and direction and felt motivated to make positive changes in their lives. |
| Azeez et al.（2022） | A complicated grief experience | Disbelief at neonatal death outcome | Disenfranchised grief: lack of social recognition and  acknowledgement | it challenging to balance expressing their grief with a desire to support others and attend to responsibilities |  |
| Kavanaugh et al.（2005） | Feeling intense emotions after the death | Parents tried to make sense of their loss and determine why it occurred | Feeling abandoned or unsupported.Seeking diversions and support | Creating and Cherishing Memories of their Infant | Contemplating future pregnancies |
| Sutan et al.（2012） | Confusion、Feeling of emptiness、Anger、Anger、Anxiety over subsequent pregnancy | Parents tried to make sense of their loss and determine why it occurred. | support during grief. | by practicing religious activities they were able to reduce the pain they suffered and make their mind more accepting of the situation. |  |
| Arach et al.（2022） | pain, confusion, facing multiple challenging roles, concern about the health of the partners, health care providers’ reaction, blame and guilt. | Women were commonly blamed by their partners and in-laws for the perinatal deaths. This could be because family members were searching for the cause of the perinatal deaths. | Family and community support |  |  |
| Horeyet al.(2012) | hazy, emotionally wrecked  My biggest fear, and that's what a lot of people would have about it, is ‘oh shit, I might have done something wrong and now I'm going to be blamed. | Because I’m not asking questions and I don’t have the what if. And even though it was unexplained I did everything I could to find out for myself. | Your doctor makes other decisions for you like .They are quite happy to make those decisions and guide you so why is suddenly when things go wrong... everyone backs off. | It was like another way of reconnecting with him. You get such a level of detail about your child that you wouldn't get in a different setting. | The autopsy did explain the death and gave the parents new information to consider when planning for another baby. |
