# Supplementary material for: Experiences and needs of family members of perinatal infant deaths: a meta-synthesis
Source: Front Public Health. 2025 Jul 1;13:1580039. doi: 10.3389/fpubh.2025.1580039 (PMC12261921; doi:10.3389/fpubh.2025.1580039)
Supplement: Supplementary file 3 [file Table_3.docx]

**Search strategies**

| Database | Search | Search terms | Results |
| --- | --- | --- | --- |
| PubMed | #1 | "Perinatal Death"[MeSH Terms] | 2313 |
|  | #2 | "perinatal death"[MeSH Terms] OR ("perinatal"[All Fields] AND "death"[All Fields]) OR "perinatal death"[All Fields] OR ("death"[All Fields] AND "perinatal"[All Fields]) OR "death perinatal"[All Fields] | 17,882 |
|  | #3 | "perinatal death"[MeSH Terms] OR ("perinatal"[All Fields] AND "death"[All Fields]) OR "perinatal death"[All Fields] OR ("deaths"[All Fields] AND "perinatal"[All Fields]) OR "deaths perinatal"[All Fields] | 20,695 |
|  | #4 | "perinatal death"[MeSH Terms] OR ("perinatal"[All Fields] AND "death"[All Fields]) OR "perinatal death"[All Fields] OR ("neonatal"[All Fields] AND "death"[All Fields]) OR "neonatal death"[All Fields] | 38298 |
|  | #5 | "perinatal death"[MeSH Terms] OR ("perinatal"[All Fields] AND "death"[All Fields]) OR "perinatal death"[All Fields] OR ("death"[All Fields] AND "neonatal"[All Fields]) OR "death neonatal"[All Fields] | 38298 |
|  | #6 | "perinatal death"[MeSH Terms] OR ("perinatal"[All Fields] AND "death"[All Fields]) OR "perinatal death"[All Fields] OR ("deaths"[All Fields] AND "neonatal"[All Fields]) OR "deaths neonatal"[All Fields] | 25982 |
|  | #7 | "perinatal death"[MeSH Terms] OR ("perinatal"[All Fields] AND "death"[All Fields]) OR "perinatal death"[All Fields] OR ("neonatal"[All Fields] AND "deaths"[All Fields]) OR "neonatal deaths"[All Fields] | 25982 |
|  | #8Combine | "Perinatal Death"[MeSH Terms] OR ("perinatal"[All Fields] AND "death"[All Fields]) OR "Perinatal Death"[All Fields] OR ("neonatal"[All Fields] AND "deaths"[All Fields]) OR "neonatal deaths"[All Fields] OR ("Perinatal Death"[MeSH Terms] OR ("perinatal"[All Fields] AND "death"[All Fields]) OR "Perinatal Death"[All Fields] OR ("deaths"[All Fields] AND "neonatal"[All Fields]) OR "deaths neonatal"[All Fields]) OR ("Perinatal Death"[MeSH Terms] OR ("perinatal"[All Fields] AND "death"[All Fields]) OR "Perinatal Death"[All Fields] OR ("death"[All Fields] AND "neonatal"[All Fields]) OR "death neonatal"[All Fields]) OR ("Perinatal Death"[MeSH Terms] OR ("perinatal"[All Fields] AND "death"[All Fields]) OR "Perinatal Death"[All Fields] OR ("neonatal"[All Fields] AND "death"[All Fields]) OR "neonatal death"[All Fields]) OR ("Perinatal Death"[MeSH Terms] OR ("perinatal"[All Fields] AND "death"[All Fields]) OR "Perinatal Death"[All Fields] OR ("deaths"[All Fields] AND "perinatal"[All Fields]) OR "deaths perinatal"[All Fields]) OR ("Perinatal Death"[MeSH Terms] OR ("perinatal"[All Fields] AND "death"[All Fields]) OR "Perinatal Death"[All Fields] OR ("death"[All Fields] AND "perinatal"[All Fields]) OR "death perinatal"[All Fields]) OR "Perinatal Death"[MeSH Terms] | 44983 |
|  | #9 | "Family"[MeSH Terms] | 384,902 |
|  | #10 | "familialities"[All Fields] OR "familiality"[All Fields] OR "familially"[All Fields] OR "familials"[All Fields] OR "familie"[All Fields] OR "family"[MeSH Terms] OR "family"[All Fields] OR "familial"[All Fields] OR "families"[All Fields] OR "family s"[All Fields] OR "familys"[All Fields] | 1,914,952 |
|  | #11 | "family"[MeSH Terms] OR "family"[All Fields] OR ("family"[All Fields] AND "life"[All Fields] AND "cycles"[All Fields]) OR "family life cycles"[All Fields] | 1,645,996 |
|  | #12 | "family"[MeSH Terms] OR "family"[All Fields] OR ("life"[All Fields] AND "cycle"[All Fields] AND "family"[All Fields]) OR "life cycle family"[All Fields] | 1,645,996 |
|  | #13 | "family"[MeSH Terms] OR "family"[All Fields] OR ("life"[All Fields] AND "cycles"[All Fields] AND "family"[All Fields]) | 1,645,996 |
|  | #14 | "family"[MeSH Terms] OR "family"[All Fields] OR ("family"[All Fields] AND "life"[All Fields] AND "cycle"[All Fields]) OR "family life cycle"[All Fields] | 1,645,996 |
|  | #15 | "family"[MeSH Terms] OR "family"[All Fields] OR ("family"[All Fields] AND "research"[All Fields]) OR "family research"[All Fields] | 1,645,996 |
|  | #16 | "family"[MeSH Terms] OR "family"[All Fields] OR ("research"[All Fields] AND "family"[All Fields]) OR "research family"[All Fields] | 1,645,996 |
|  | #17 | "family"[MeSH Terms] OR "family"[All Fields] OR ("family"[All Fields] AND "members"[All Fields]) OR "family members"[All Fields] | 1,645,996 |
|  | #18 | "family"[MeSH Terms] OR "family"[All Fields] OR ("family"[All Fields] AND "member"[All Fields]) OR "family member"[All Fields] | 1,645,996 |
|  | #19 | "family"[MeSH Terms] OR "family"[All Fields] OR "filiation"[All Fields] | 1,646,186 |
|  | #20 | "family"[MeSH Terms] OR "family"[All Fields] OR ("kinship"[All Fields] AND "networks"[All Fields]) OR "kinship networks"[All Fields] | 1,646,163 |
|  | #21 | "family"[MeSH Terms] OR "family"[All Fields] OR ("kinship"[All Fields] AND "network"[All Fields]) OR "kinship network"[All Fields] | 1,646,185 |
|  | #22 | "family"[MeSH Terms] OR "family"[All Fields] OR ("network"[All Fields] AND "kinship"[All Fields]) OR "network kinship"[All Fields] | 1,646,185 |
|  | #23 | "family"[MeSH Terms] OR "family"[All Fields] OR ("networks"[All Fields] AND "kinship"[All Fields]) OR "networks kinship"[All Fields] | 1,646,163 |
|  | #24 | "family"[MeSH Terms] OR "family"[All Fields] OR "relative"[All Fields] OR "relatives"[All Fields] OR "relative s"[All Fields] OR "relatively"[All Fields] | 3,308,469 |
|  | #25 | "Family"[MeSH Terms] OR "Family"[All Fields] OR "relative"[All Fields] OR "relatives"[All Fields] OR "relative s"[All Fields] OR "relatively"[All Fields] OR ("Family"[MeSH Terms] OR "Family"[All Fields] OR ("networks"[All Fields] AND "kinship"[All Fields]) OR "networks kinship"[All Fields]) OR ("Family"[MeSH Terms] OR "Family"[All Fields] OR ("network"[All Fields] AND "kinship"[All Fields]) OR "network kinship"[All Fields]) OR ("Family"[MeSH Terms] OR "Family"[All Fields] OR ("kinship"[All Fields] AND "network"[All Fields]) OR "kinship network"[All Fields]) OR ("Family"[MeSH Terms] OR "Family"[All Fields] OR ("kinship"[All Fields] AND "networks"[All Fields]) OR "kinship networks"[All Fields]) OR ("Family"[MeSH Terms] OR "Family"[All Fields] OR "filiation"[All Fields]) OR ("Family"[MeSH Terms] OR "Family"[All Fields] OR ("Family"[All Fields] AND "member"[All Fields]) OR "family member"[All Fields]) OR ("Family"[MeSH Terms] OR "Family"[All Fields] OR ("Family"[All Fields] AND "members"[All Fields]) OR "family members"[All Fields]) OR ("Family"[MeSH Terms] OR "Family"[All Fields] OR ("research"[All Fields] AND "Family"[All Fields]) OR "research family"[All Fields]) OR ("Family"[MeSH Terms] OR "Family"[All Fields] OR ("Family"[All Fields] AND "research"[All Fields]) OR "family research"[All Fields]) OR ("Family"[MeSH Terms] OR "Family"[All Fields] OR ("Family"[All Fields] AND "life"[All Fields] AND "cycle"[All Fields]) OR "family life cycle"[All Fields]) OR ("Family"[MeSH Terms] OR "Family"[All Fields] OR ("life"[All Fields] AND "cycles"[All Fields] AND "Family"[All Fields])) OR ("Family"[MeSH Terms] OR "Family"[All Fields] OR ("life"[All Fields] AND "cycle"[All Fields] AND "Family"[All Fields]) OR "life cycle family"[All Fields]) OR ("Family"[MeSH Terms] OR "Family"[All Fields] OR ("Family"[All Fields] AND "life"[All Fields] AND "cycles"[All Fields]) OR "family life cycles"[All Fields]) OR ("familialities"[All Fields] OR "familiality"[All Fields] OR "familially"[All Fields] OR "familials"[All Fields] OR "familie"[All Fields] OR "Family"[MeSH Terms] OR "Family"[All Fields] OR "familial"[All Fields] OR "families"[All Fields] OR "family s"[All Fields] OR "familys"[All Fields]) OR "Family"[MeSH Terms] | 3,556,793 |
|  | #26 | "Qualitative Research"[MeSH Terms] | 96,818 |
|  | #27 | "Qualitative Research"[MeSH Terms] AND ("Family"[MeSH Terms] OR "Family"[All Fields] OR "relative"[All Fields] OR "relatives"[All Fields] OR "relative s"[All Fields] OR "relatively"[All Fields] OR ("Family"[MeSH Terms] OR "Family"[All Fields] OR ("networks"[All Fields] AND "kinship"[All Fields]) OR "networks kinship"[All Fields]) OR ("Family"[MeSH Terms] OR "Family"[All Fields] OR ("network"[All Fields] AND "kinship"[All Fields]) OR "network kinship"[All Fields]) OR ("Family"[MeSH Terms] OR "Family"[All Fields] OR ("kinship"[All Fields] AND "network"[All Fields]) OR "kinship network"[All Fields]) OR ("Family"[MeSH Terms] OR "Family"[All Fields] OR ("kinship"[All Fields] AND "networks"[All Fields]) OR "kinship networks"[All Fields]) OR ("Family"[MeSH Terms] OR "Family"[All Fields] OR "filiation"[All Fields]) OR ("Family"[MeSH Terms] OR "Family"[All Fields] OR ("Family"[All Fields] AND "member"[All Fields]) OR "family member"[All Fields]) OR ("Family"[MeSH Terms] OR "Family"[All Fields] OR ("Family"[All Fields] AND "members"[All Fields]) OR "family members"[All Fields]) OR ("Family"[MeSH Terms] OR "Family"[All Fields] OR ("research"[All Fields] AND "Family"[All Fields]) OR "research family"[All Fields]) OR ("Family"[MeSH Terms] OR "Family"[All Fields] OR ("Family"[All Fields] AND "research"[All Fields]) OR "family research"[All Fields]) OR ("Family"[MeSH Terms] OR "Family"[All Fields] OR ("Family"[All Fields] AND "life"[All Fields] AND "cycle"[All Fields]) OR "family life cycle"[All Fields]) OR ("Family"[MeSH Terms] OR "Family"[All Fields] OR ("life"[All Fields] AND "cycles"[All Fields] AND "Family"[All Fields])) OR ("Family"[MeSH Terms] OR "Family"[All Fields] OR ("life"[All Fields] AND "cycle"[All Fields] AND "Family"[All Fields]) OR "life cycle family"[All Fields]) OR ("Family"[MeSH Terms] OR "Family"[All Fields] OR ("Family"[All Fields] AND "life"[All Fields] AND "cycles"[All Fields]) OR "family life cycles"[All Fields]) OR ("familialities"[All Fields] OR "familiality"[All Fields] OR "familially"[All Fields] OR "familials"[All Fields] OR "familie"[All Fields] OR "Family"[MeSH Terms] OR "Family"[All Fields] OR "familial"[All Fields] OR "families"[All Fields] OR "family s"[All Fields] OR "familys"[All Fields]) OR "Family"[MeSH Terms]) AND ("Perinatal Death"[MeSH Terms] OR ("perinatal"[All Fields] AND "death"[All Fields]) OR "Perinatal Death"[All Fields] OR ("neonatal"[All Fields] AND "deaths"[All Fields]) OR "neonatal deaths"[All Fields] OR ("Perinatal Death"[MeSH Terms] OR ("perinatal"[All Fields] AND "death"[All Fields]) OR "Perinatal Death"[All Fields] OR ("deaths"[All Fields] AND "neonatal"[All Fields]) OR "deaths neonatal"[All Fields]) OR ("Perinatal Death"[MeSH Terms] OR ("perinatal"[All Fields] AND "death"[All Fields]) OR "Perinatal Death"[All Fields] OR ("death"[All Fields] AND "neonatal"[All Fields]) OR "death neonatal"[All Fields]) OR ("Perinatal Death"[MeSH Terms] OR ("perinatal"[All Fields] AND "death"[All Fields]) OR "Perinatal Death"[All Fields] OR ("neonatal"[All Fields] AND "death"[All Fields]) OR "neonatal death"[All Fields]) OR ("Perinatal Death"[MeSH Terms] OR ("perinatal"[All Fields] AND "death"[All Fields]) OR "Perinatal Death"[All Fields] OR ("deaths"[All Fields] AND "perinatal"[All Fields]) OR "deaths perinatal"[All Fields]) OR ("Perinatal Death"[MeSH Terms] OR ("perinatal"[All Fields] AND "death"[All Fields]) OR "Perinatal Death"[All Fields] OR ("death"[All Fields] AND "perinatal"[All Fields]) OR "death perinatal"[All Fields]) OR "Perinatal Death"[MeSH Terms]) | 191 |
| Web of science | #1 | ALL=(Perinatal Death) | 16,288 |
|  | #2 | ALL=(Perinatal Deaths) | 16247 |
|  | #3 | ALL=(Neonatal Death) | 30155 |
|  | #4 | ALL=(Neonatal Deaths) | 29985 |
|  | **#5 Combine** | **#1 OR #2 OR #3 OR #4** | **[38,127](https://www-webofscience-com-ssl.proxy.cuk.ac.kr/wos/woscc/summary/ab14ee63-ee45-401e-a23d-91131e7c625f-01376080d9/relevance/1)** |
|  | #6 | ALL=(Family) | [1,873,435](https://www-webofscience-com-ssl.proxy.cuk.ac.kr/wos/woscc/summary/0b9405ed-de49-4154-857c-4e6fecc2a5d6-0137609b98/relevance/1) |
|  | #7 | ALL=(Families) | [1,562,954](https://www-webofscience-com-ssl.proxy.cuk.ac.kr/wos/woscc/summary/cd053ca6-1566-495c-9065-80582258ee7d-013760a614/relevance/1) |
|  | #8 | ALL=(Family Life Cycles) | [14,566](https://www-webofscience-com-ssl.proxy.cuk.ac.kr/wos/woscc/summary/77d48037-29c5-4ad6-b2d9-25b455feffcc-013760ae2b/relevance/1) |
|  | #9 | ALL=(Family Life Cycle) | [14,879](https://www-webofscience-com-ssl.proxy.cuk.ac.kr/wos/woscc/summary/6a229baf-d03b-4a6f-ac86-b632b3a0b312-013760d3cc/relevance/1) |
|  | #10 | ALL=(Family Research) | [986,454](https://www-webofscience-com-ssl.proxy.cuk.ac.kr/wos/woscc/summary/dc15a1ee-9287-4163-a9f8-6295be1aa88a-013760e2fe/relevance/1) |
|  | #11 | ALL=(Family Members) | [329,769](https://www-webofscience-com-ssl.proxy.cuk.ac.kr/wos/woscc/summary/b96a58b0-2ec4-4ecc-93ea-942dc72b52b6-013760eb95/relevance/1) |
|  | #12 | ALL=(Family Member) | [314,072](https://www-webofscience-com-ssl.proxy.cuk.ac.kr/wos/woscc/summary/747c9613-36b4-4932-b7af-6cbf6c5f53ed-013760f2e0/relevance/1) |
|  | #13 | ALL=(Filiation) | 340 |
|  | #14 | ALL=(Kinship Networks) | 799 |
|  | #15 | ALL=(Kinship Network) | 879 |
|  | #16 | ALL=(Relatives) | [1,979,205](https://www-webofscience-com-ssl.proxy.cuk.ac.kr/wos/woscc/summary/c657ad94-ebdf-4c37-bea3-574a4558f15a-013761178c/relevance/1) |
|  | **#17Combine** | **#16 OR #15 OR #14 OR #13 OR #12 OR #11 OR #10 OR #9 OR #8 OR #7 OR #6** | **[3,794,984](https://www-webofscience-com-ssl.proxy.cuk.ac.kr/wos/woscc/summary/01595445-94ea-40be-9da4-6b4a10844c56-01376244be/relevance/1)** |
|  | #18 | ALL=(qualitative research) | [296,658](https://www-webofscience-com-ssl.proxy.cuk.ac.kr/wos/woscc/summary/1af8a9a4-5b0d-4dfc-a2ed-4c1d66bc1e25-0137627caf/relevance/1) |
|  | **#19Combine** | **#18 AND #17 AND #5** | **[192](https://www-webofscience-com-ssl.proxy.cuk.ac.kr/wos/woscc/summary/6476e488-1e52-43f4-9fd0-dcfa334e1acb-0137628d14/relevance/1)** |
| Embase | #1 | 'perinatal death' | 8,023 |
|  | #2 | 'perinatal deaths' | 2,856 |
|  | #3 | 'neonatal death' | 9,246 |
|  | #4 | 'neonatal deaths' | 5,832 |
|  | **#5 Combine** | **#1 OR #2 OR #3 OR #4** | 21,865 |
|  | #6 | 'Family' | 1,816,725 |
|  | #7 | 'Families' | 399,734 |
|  | #8 | 'Family Life Cycle' | 331 |
|  | #9 | 'Family Research' | [680,903](https://www-embase-com-443.webvpn.cams.tsgvip.top/) |
|  | #10 | 'Family Member' | 160,548 |
|  | #11 | 'Filiation' | 519 |
|  | #12 | 'Kinship Network' | 43 |
|  | #13 | 'Relatives' | 96,940 |
|  | **#14 Combine** | **#6 or #7 or #8 or #9 or #10 or #11 or #12 or #13** | **2,083,325** |
|  | #15 | 'qualitative research' | 154,020 |
|  | **#16 Combine** | **#5 AND #14 AND #15** | **86** |
| CINAHL | S1 | TX perinatal death | 11,606 |
|  | S2 | TX neonatal death | 5,941 |
|  | **S3 Combine** | **S1 OR S2** | **15,807** |
|  | S4 | TX Family | 855,164 |
|  | S5 | TX Family Life Cycle | 599 |
|  | S6 | TX Family Research | 3,920 |
|  | S7 | TX Family Member | 115,555 |
|  | S8 | TX Filiation | 185 |
|  | S9 | TX Kinship Network | 422 |
|  | S10 | TX Relatives | 369,149 |
|  | **S11 Combine** | **S4 OR S5 OR S6 OR S7 OR S8 OR S9 OR S10** | **1,103,077** |
|  | S12 | TX qualitative research | 19,693 |
|  | **S13 Combine** | **S3 AND S11 AND S12** | **27** |
