# Supplementary material for: Experiences and needs of family members of perinatal infant deaths: a meta-synthesis
Source: Front Public Health. 2025 Jul 1;13:1580039. doi: 10.3389/fpubh.2025.1580039 (PMC12261921; doi:10.3389/fpubh.2025.1580039)
Supplement: Supplementary file 4 [file Table_4.docx]

**List of excluded studies**

| No. | Author (Year) | Title |
| --- | --- | --- |
| 1 | Zheng et al.(2024) | The influence of culture and spirituality on maternal grief following stillbirth in China: A qualitative study |
| 2 | Giti et al.(2024) | Desired Care for Perinatal Bereavement: Meeting the Needs of Mothers After Discharge From the Hospital-a Qualitative Study |
| 3 | Gill et al.(2024) | Experiences and impacts of psychological support following adverse neonatal experiences or perinatal loss: a qualitative analysis |
| 4 | Kuforiji et al.(2024) | An exploration of women's lived experiences of care and support following perinatal death in South-Western Nigeria: A hermeneutic phenomenological study |
| 5 | Gillian et al.(2024) | Situational Analysis on the Impact of Perinatal Deaths Among Bereaved Families in Ghana |
| 6 | Debbie et al.(2023) | Providing Lactation Care Following Stillbirth, Neonatal and Infant Death: Learning from Bereaved Parents |
| 7 | Anna et al.(2022) | Your heart keeps bleeding”: lived experiences of parents with a perinatal death in Northern Uganda |
| 8 | Tracey A et al.(2022) | Better maternity care pathways in pregnancies after stillbirth or neonatal death: a feasibility study |
| 9 | Thomas et al.(2022) | Culturally and linguistically diverse men's experiences of support following perinatal death: A qualitative study |
| 10 | Arach(2022) | “Your heart keeps bleeding”: lived experiences of parents with a perinatal death in Northern Uganda |
| 11 | Azeez et al.(2022) | Overwhelming and unjust: A qualitative study of fathers' experiences of grief following neonatal death |
| 12 | Heloisa et al.(2021) | The perinatal bereavement project: development and evaluation of supportive guidelines for families experiencing stillbirth and neonatal death in Southeast Brazil-a quasi-experimental before-and-after study |
| 13 | Sergio et al.(2021) | Preliminary fndings on the experiences of care for parents who sufered perinatal bereavement during the COVID-19 pandemic |
| 14 | Willyane et al.(2021) | Experience of hope: An exploratory research with bereaved mothers following perinatal death |
| 15 | Shazleen et al.(2021) | Australian fathers' experiences of support following neonatal death a need for better access to diverse support options |
| 16 | Redshaw et al.(2021) | 'This is time we'll never get back': a qualitative study of mothers' experiences of care associated with neonatal death |
| 17 | Nadin et al.(2021) | Informing mothers of neonatal death and the need for family‐centered bereavement care: A phenomenological qualitative study |
| 18 | Manoja et al.(2021) | Grief reaction and psychosocial impacts of child death and stillbirth on bereaved North Indian parents: A qualitative study |
| 19 | Cayetano et al.(2021) | Impact of Perinatal Death on the Social and Family Context of the Parents |
| 20 | Rebecca et al.(2020) | A qualitative study exploring the experiences of bereavement after stillbirth in pakistani, bangladeshi and white british mothers living in luton, UK. |
| 21 | [Jamil](https://pubmed.ncbi.nlm.nih.gov/?size=200&term=Ahmed+J&cauthor_id=33022424) et al.(2020) | Traditional practices during pregnancy and birth, and perceptions of perinatal losses in women of rural Pakistan |
| 22 | Smith et al.(2020) | Parents’ experiences of care following the loss of a baby at the margins between miscarriage, stillbirth and neonatal death: a UK qualitative study |
| 23 | Jami et al.(2020) | Exploring women’s experience of healthcare use during pregnancy and childbirth to understand factors contributing to perinatal  deaths in Pakistan: A qualitative study |
| 24 | Carmen et al.(2019) | Parents’ Wishes for what they had or had not done and their Coping after their Infant’s or Child’s NICU/PICU/ED Death |
| 25 | Jo Watson et al.(2019) | Pregnancy and infant loss: a survey of families’ experiences in Ontario Canada |
| 26 | Camacho et al.(2019) | Experience of parents who have suffered a perinatal death in two Spanish hospitals: A qualitative study |
| 27 | Lizcano Pabón et al.(2019) | Experience of Perinatal Death From the Father's Perspective |
| 28 | Pamela et al.(2017) | Not just a normal mum”: a qualitative investigation of a support service for women who are pregnant subsequent to perinatal loss |
| 29 | Marina et al.(2017) | Communication of death and grief support to the women who have lost a newborn child |
| 30 | [Aiya](https://pubmed.ncbi.nlm.nih.gov/?size=200&term=Golan+A&cauthor_id=29206946) et al.(2016) | Meaning Reconstruction among Women following Stillbirth: A Loss Fraught with Ambiguity and Doubt |
| 31 | Anna et al.(2016) | They Say I Should not Think About It:” A Qualitative Study Exploring the Experience of Infant Loss for Bereaved Mothers in Kumasi, Ghana |
| 32 | Micah et al.(2015) | Caring for families experiencing stillbirth: Evidence-based guidance for maternity care providers |
| 33 | [Judy](https://pubmed.ncbi.nlm.nih.gov/?size=200&term=Richards+J&cauthor_id=26134115)et al.(2015) | Mothers' perspectives on the perinatal loss of a co-twin: a qualitative study |
| 34 | Narges et al.(2015) | Spiritual Needs of Families with Bereavement and Loss of an Infant in Neonatal Intensive Care Unit: A Qualitative Study |
| 35 | Kirsty et al.(2014) | Mothers’ experience of their contact with their stillborn infant: An interpretative phenomenological analysis |
| 36 | Soo et al.(2013) | Bereaved parents' experience of stillbirth in UK hospitals a qualitative interview study |
| 37 | Maureen et al.(2012) | Silent loss and the clinical encounter: Parents’ and physicians’ experiences of stillbirth–a qualitative analysis |
| 38 | Sutan et al.（2012） | Psychosocial impact of perinatal loss among Muslim women |
| 39 | Jane et al.(2011) | Born after infant loss: The experiences of subsequent children |
| 40 | Laurence et al.(2011) | Living with a crucial decision: a qualitative study of parental narratives three years after the loss of their newborn in the NICU |
| 41 | Einaudi et al.(2010) | Parental experience following perinatal death: Exploring the issues to make progress |
| 42 | Yamazaki et al.(2010) | Living With Stillborn Babies as Family Members: Japanese Women Who Experienced Intrauterine Fetal Death After 28 Weeks Gestation |
| 43 | Kavanaugh et al.(2005) | Perinatal loss in low-income African American parents |
| 44 | Horeyet al.(2012) | Decision influences and aftermath: parents, stillbirth and autopsy |
